# Tipping the Balance: Context and Cell-Specific Regulation of MMPs and TIMPs in Liver Fibrosis

**DOI:** 10.3390/biom16101513

**Published:** 2026-10-18

**Authors:** Stephen W. Harris, Purnima Tyagi, Robert C. Huebert

**Affiliations:** 1 Department of Internal Medicine, Wright State University, 30 E. Apple St., Suite 3290, Dayton, OH 45409, USA; stephen.harris@wright.edu; 2 Division of Gastroenterology and Hepatology, Mayo Clinic, 200 1st Street SW, Rochester, MN 55905, USA; 3 Gastroenterology Research Unit, Mayo Clinic, 200 1st Street SW, Rochester, MN 55905, USA; 4 Mayo Clinic Center for Cell Signaling in Gastroenterology, Mayo Clinic and Foundation, 200 1st Street SW, Rochester, MN 55905, USA

**Keywords:** MMPs, TIMPs, hepatic fibrosis, fibrosis, HSCs, fibroblasts, collagen

## Abstract

Chronic liver disease represents a substantial global health burden, yet effective anti-fibrotic therapies remain elusive, largely due to an incomplete understanding of extracellular matrix (ECM) remodeling. While historical research prioritized the role of matrix metalloproteinase (MMP) deficiency in fibrogenesis, clinical outcomes indicate that broad-spectrum MMP modulation is paradoxically ineffective. This review synthesizes current evidence to demonstrate that hepatic fibrosis is driven by a complex regulatory imbalance wherein tissue inhibitors of metalloproteinases (TIMPs) sustain a pathological microenvironment. We highlight that TIMPs facilitate fibrogenesis through two primary mechanisms: the inhibition of MMP-mediated ECM degradation and the suppression of apoptosis in activated hepatic stellate cells (HSCs), a process exacerbated by mechanotransduction-driven feedback loops. Importantly, we address the functional redundancy within the TIMP family, as evidenced by the progression of fibrosis in TIMP-1-deficient models, which challenges traditional linear regulatory paradigms. Ultimately, we propose that the future of fibrosis treatment necessitates a shift from broad-spectrum enzyme modulation toward precision molecular strategies, including small-molecule inhibition, antisense oligonucleotides, and epigenetic reprogramming aimed at restoring the physiological MMP/TIMP equilibrium within the fibrotic niche.

## 1. Introduction

Liver diseases are a major public health concern, contributing to approximately 2 million deaths globally each year [1].

According to the Global Burden of Disease (GBD) 2019 study, approximately 1.26 million individuals died from cirrhosis and other chronic liver diseases in 2019 [2]. Approximately 30% of the U.S. population is at risk of developing metabolic disease-associated steatohepatitis (MASH), reflecting a growing public health burden. While the expanding use of metabolic therapies, such as GLP-1 receptor agonists, shows promise in attenuating upstream steatohepatitis and metabolic drivers, therapies that directly promote the resolution of an established, mature fibrotic matrix remain elusive despite the enormous clinical burden. Thus, elucidating matrix degradation pathways and identifying direct anti-fibrotic targets remain an urgent priority.

Liver fibrogenesis is a dynamic process characterized by excessive extracellular matrix (ECM) accumulation and impaired matrix degradation mediated by metalloproteinases. These changes typically arise in response to chronic liver injury caused by diverse insults. The principal cellular mediators of ECM production are activated portal fibroblasts (PFs) and hepatic stellate cells (HSCs), which transdifferentiate into contractile, fibrogenic, and proinflammatory myofibroblasts [3]. In this review, we summarize both historical and current research on the roles of tissue inhibitors of matrix metalloproteinases (TIMPs) in fibrogenesis and fibrolysis.

Under physiological conditions, matrix metalloproteinases (MMPs) and TIMPs act in concert to tightly regulate ECM generation and turnover. This intricate system is dysregulated when the liver is acutely or chronically damaged via conditions such as alcohol-related liver disease, MASH, iron overload, or chronic inflammation due to viral hepatitis. Liver fibroblasts including PFs and HSCs become activated and alter ECM composition through the production and secretion of collagen and other components of the hepatic matrisome [4,5]. The resulting accumulation of dense fibrotic scar tissue underlies progressive liver fibrosis, which can ultimately lead to cirrhosis. Although advanced fibrosis and cirrhosis were once considered irreversible, recent studies have demonstrated potential for some spontaneous resolution if the initial insult is halted (e.g., abstinence in alcohol-related liver disease or clearance of viral hepatitis). MMP concentrations fluctuate throughout fibrogenesis and fibrolysis. Their enzymatic degradation of ECM components, including collagen, is pivotal to both the development and resolution of fibrosis. Conversely, TIMPs may promote the persistence of fibrosis by inhibiting ECM degradation and by preventing apoptosis of activated fibroblasts responsible for scar deposition. Because ECM accumulation is governed by the equilibrium between MMP proteolytic activity and TIMP inhibition, shifting this axis dictates whether chronic liver injury progresses to cirrhosis or undergoes resolution. This review will explore the roles and interactions of TIMPs with key cellular and molecular mediators of hepatic fibrosis, including PFs, HSCs, MMPs, and various ECM proteins such as collagens and fibrins.

## 2. Matrix Metallo-Proteinases (MMPs)

The matrix metalloproteinase (MMP) family comprises 23 members, including collagenases, stromelysins, matrilysins, gelatinases, metalloelastase, and membrane-type MMPs. Collectively known as matrixins, these calcium-dependent, zinc-containing endopeptidases are essential for normal tissue homeostasis, remodeling, and repair. MMPs may be either secreted or membrane-bound. Most are synthesized as inactive zymogens (pro-MMPs) that require proteolytic cleavage for activation. For example, TIMP-2 participates in the cell-surface activation of pro-MMP-2 by forming a trimolecular complex with MT1-MMP (MMP-14), which presents pro-MMP-2 for cleavage by an adjacent TIMP-2-free MT1-MMP molecule [6]. MMPs’ expression is often upregulated during acute injury or inflammation, where their proteolytic activity clears cellular debris and degraded matrix, creating space for tissue regeneration. Spatial expression studies have characterized MMP localization during hepatic fibrosis. Al-Humadi et al. [7] demonstrated via immunohistochemistry that MMP-2 is expressed in periportal regions, hepatocytes, stromal cells, cholangiocytes, and around central veins. Similarly, MMP-9 is expressed in hepatocytes, hepatic fibroblasts, and inflammatory mononuclear cells near portal tracts and central veins.

While there are many MMPs, not all of them have a significant role in fibrogenesis. MMP-1, MMP-2, MMP-9, MMP-12, MMP-13, and MMP-14 have been identified in several studies as possible contributors to fibrosis. Yang et al. observed MMP-2 and MMP-9 overexpression in a model of radiation-induced lung inflammation [8]. These enzymes target collagen IV, a principal component of basement membranes, thereby altering tissue integrity. However, during fibrosis, MMP-9 activity decreased concurrently with TIMP upregulation, suggesting that reduced MMP-mediated ECM degradation contributes to fibrotic progression.

Similar findings have been reported in hepatic fibrosis models. Al-Humadi et al. [7] showed increased MMP-2 and MMP-9 expression in thioacetamide (TAA)-induced fibrosis, and Hemmann et al. [9] identified MMP-2 and MMP-14 expression in activated HSCs following hepatic injury. Their data further indicated that MMP-9-mediated degradation of type I collagen may be critical for fibrolysis. Collectively, these findings suggest that MMP-9 exerts biphasic effects during fibrosis. In early injury, its expression reflects acute matrix remodeling, while, later, downregulation may lead to insufficient ECM degradation and fibrotic accumulation. Reactivation of MMP-9 during recovery could, therefore, facilitate fibrolysis and fibrosis resolution.

Other MMPs, including MMP-13 and MMP-14, are upregulated during fibrosis resolution [10]. Following injury cessation, restorative macrophages accumulate and promote HSC apoptosis while secreting MMP-12 and MMP-13, supporting a pro-resolutive role for these enzymes.

MMP activity is also regulated by matrix stiffness and TIMP expression. Lachowski et al. [11] demonstrated that fibrotic rigidity suppresses MMP-9 activity, whereas TIMP-1 knockdown enhances it, highlighting a mechanical and biochemical feedback loop governing MMP function. Thus, increased matrix rigidity and elevated TIMP-1 expression may synergistically inhibit MMP-9, promoting pathologic ECM accumulation. Consistently, in hepatocellular carcinoma, which commonly arises in fibrotic tissue, MMP-9 expression is suppressed while ECM protein expression increases. Others have previously reported that HSC levels of MMP-9 were upregulated, indicating that, even though HSCs may still be secreting MMP-9, other factors in the liver may stop secreting MMP-9 or may organize its removal from the system as a whole [12,13]. Overall, these findings indicate that, while MMPs are upregulated in early fibrosis, their global hepatic expression declines as disease progresses, shifting toward matrix-stabilizing protein dominance.

**Figure 1:**
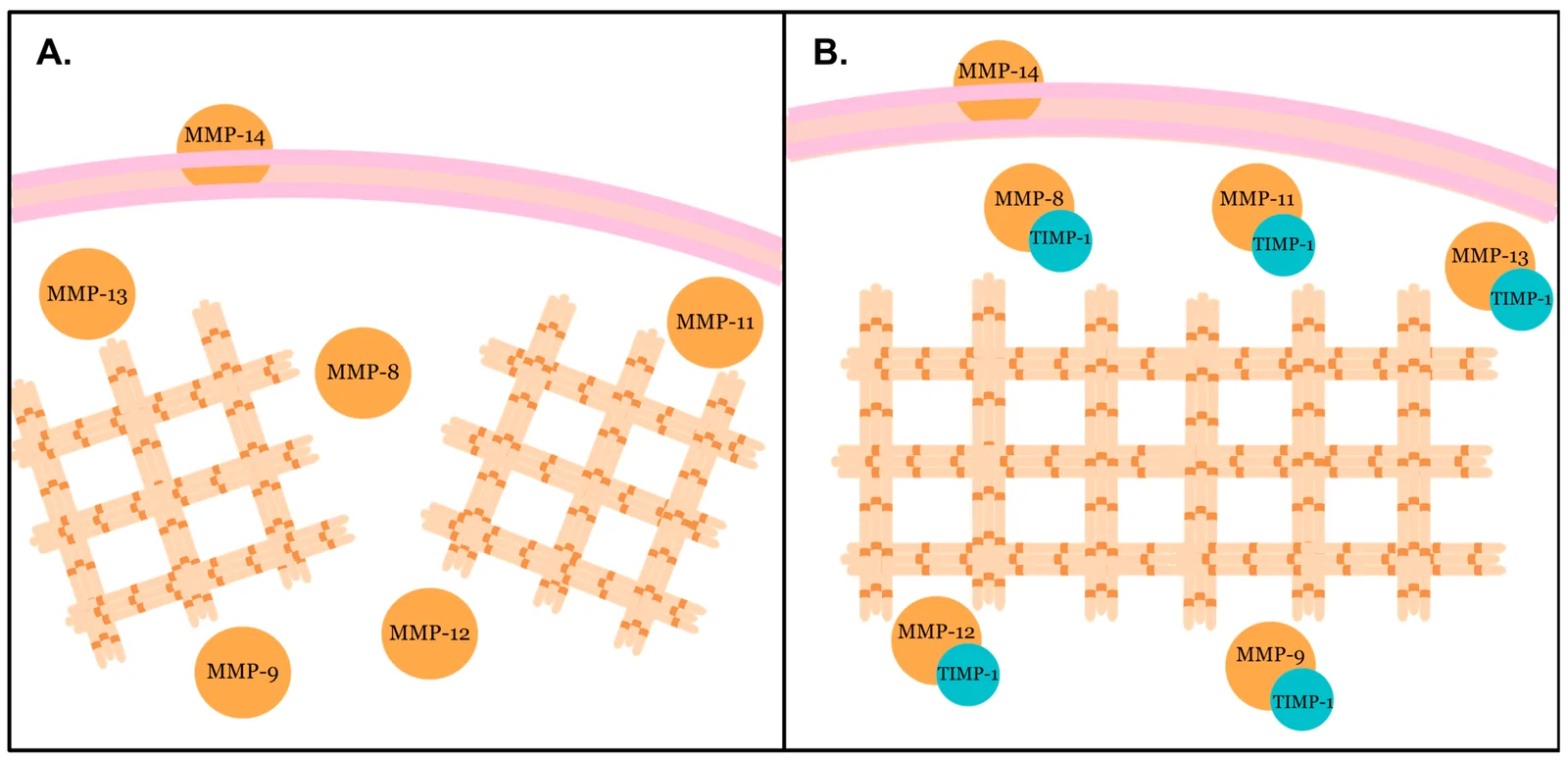
Regulation of extracellular matrix turnover by matrix metalloproteinases (MMPs) and tissue inhibitors of metalloproteinases (TIMPs). (**A**) Under physiological or active remodeling conditions, active collagenases and gelatinases (MMP-8, MMP-9, MMP-11, MMP-12, MMP-13, and membrane-bound MMP-14) degrade fibrillar collagen networks to promote fibrolysis. (**B**) During pathological fibrogenesis, upregulated TIMPs (e.g., TIMP-1) bind to active MMPs in a 1:1 stoichiometric complex, inhibiting catalytic matrix degradation and promoting excessive collagen accumulation. [4,5,6,8,17].

Supporting this concept, Fallowfield et al. [14] and Uchinami et al. [15] have shown that several MMPs are upregulated during resolution. This upregulation was also shown to be relative to TIMP expression [16] as evidenced in models of alcohol-induced fibrosis. Likewise, Zhang, M. et al. [17] found that MMP-2 and MMP-9 concentrations were decreased during fibrosis and that these concentrations were significantly restored in their treatment model. Thus, effective fibrolysis requires MMP expression sufficient to overcome TIMP-mediated inhibition, emphasizing the importance of the MMP–TIMP balance in both fibrosis progression and resolution (see Figure 1).

Because the anti-fibrotic capacity of these proteases is ultimately constrained in vivo by endogenous inhibitors, understanding matrix turnover requires examining the regulatory counterweight: the TIMP family.

## 3. Tissue Inhibitors of Matrix Metalloproteinases (TIMPs)—Their Interactions with MMPs and Their Role in Fibrogenesis

There are 4 known TIMPs that have a variety of effects on fibrosis. Each TIMP can bind to and inhibit all 23 known MMPs, with some variation in binding affinity (see Table 1 below). These inhibitors have a low molecular weight of around 20kDa. Mechanistically, they inhibit MMPs by binding directly to the active site in a 1:1 stoichiometric ratio [18]. Some TIMPs have a binding affinity for A disintegrin and metalloproteinase (ADAM) proteins that share some attributes with MMPs. TIMPs also have effects on the life cycles of multiple cell types in fibrosis [19].

TIMP-1 and TIMP-2 expression is primarily localized to hepatic fibroblasts and hepatocytes adjacent to fibrotic septa and also in regions surrounding the central vein [7]. Van Riet et al. [35] have shown that cultures containing liver sinusoidal endothelial cells also have markedly increased secretion of TIMP-1. However, Liu et al. [36] reported that TIMP-2 mRNA expression was not found in hepatocytes but was found in activated HSCs and Kupffer cells. TIMP-1 significantly increases in the serum and in the liver during the development of fibrosis [37]. TIMP-1 and TIMP-2 specifically have been heavily implicated in hepatic fibrogenesis. Benyon et al. [38] reported anywhere from a 367% to a 724% upregulation in the mRNA of TIMP-1 in fibrotic livers. It was concluded that TIMP-1 concentration increases in liver disease and causes the cessation of ECM degradation. Building on these early findings, Yang et al. [8] demonstrated significant upregulation of TIMP-1, TIMP-2, and TIMP-3 in a model of radiation-induced lung fibrosis. More recently, Zhang et al. [17] reported significant increases in both TIMP-1 and TIMP-2 levels in a CCl_4_-induced model of hepatic fibrosis. In FFA-induced fibrotic spheroid models, Van Riet et al. [35] found elevated expression of TIMP-1 and TIMP-2, which corresponded with increased deposition of fibrotic collagens, including procollagen and mature type I collagen, an effect that was not replicated in siRNA TIMP-1-silenced spheroid models. In these silenced models, ARP–100 (a known MMP inhibitor), restored deposition of fibrotic collagens, further suggesting that TIMP-driven suppression of MMPs is a key determinant of ECM remodeling and fibrosis.

Beyond suppressing collagen turnover, TIMP-1 may also affect the motility of activated HSCs during fibrogenesis and fibrotic maintenance. Ramenzi-Moghadam et al. [39] reported that TIMP-1 secretion reduces HSC migratory capacity. This observation suggests that, once HSCs have migrated to sites of hepatic injury, further migration may be unnecessary. In this context, sustained TIMP-1 secretion may limit the recruitment of additional HSCs, thereby spatially restricting fibrotic activity to established regions of damage.

TIMP-1 influences the life cycle of both HSCs and hepatocytes. Murphy et al. [40] showed that TIMP-1 is specifically involved in the direct inhibition of HSC apoptosis. While TIMPs may have a positive effect on the lifespan of the HSCs, Hemmann et al. [9] showed that the lack of TIMP-1 caused an increase in hepatocyte proliferation. The authors proposed that this effect may be mediated by enhanced MMP activity, leading to reduced ECM density, increased physical space for hepatocyte expansion, and the release of ECM-bound pro-hepatocyte growth factors. Evaluation of livers at distinct stages of fibrotic development [41], showed increased TIMP-1 staining and an increased concentration of TIMP-1 producing HSCs and LSECs in MASH/MAFLD livers, with particular prevalence in the perivenous regions of large vessels, an area known to be particularly susceptible to MASH/MAFLD-related steatosis.

TIMPs have been shown to have notable interplay with other inflammatory markers in addition to their involvement in fibrosis through MMPs. Huang et al. [42] have shown that TIMPs suppress microRNA-145 in activated HSCs, thereby alleviating the repression of downstream targets such as friend leukemia virus integration-1 (Fli-1). This resulted in increased MCP-1 secretion by these HSCs, recruiting monocytes and macrophages to the liver where they secreted active inflammatory cytokines such as TGF beta. Silencing TIMP-1 using siRNA in carbon tetrachloride-exposed rats prevented the induction of fibrosis and was associated with reduced Fli-1, MCP-1, and alpha smooth muscle actin (α-SMA) levels, along with markedly decreased monocyte and macrophage recruitment.

Similar findings have also been observed in viral models of fibrosis. Zhu et al. [43] found that HSCs exposed to exosomes derived from HBV-infected hepatocytes triggered a sharp increase in TIMP-1 presentation, along with other fibrotic markers such as Col1A and α-SMA. Following treatment of HBV with entacavir or HBx knockdown, TIMP-1 levels returned to baseline alongside fibrotic markers, further confirming the involvement of TIMP-1 in the development and progression of fibrosis in this viral model.

Upstream regulators of TIMPs also play important roles in fibrosis progression across diverse pathological settings. Liu et al. [44] investigated the activity of spleen tyrosine kinase (SYK) and SH3 domain-binding protein 2 (SH3BP2, a downstream adapter and signal amplifier of SYK) in the mouse model of liver fibrosis. In this process, paeoniflorin (PF), an SYK inhibitor, decreased not only SYK levels but also TIMP-1, α-SMA and other inflammatory markers such as TLR and IL-6. However when hepatocyte cultures were transfected with SYK-overexpressing plasmids the anti-inflammatory effects of PF were largely abolished, and the expression levels of TIMP-1, α-SMA, TLR and IL-6 returned to pre-treatment levels. These findings suggest that targeting upstream regulators such as SYK can suppress TIMP-1 expression while simultaneously attenuating inflammatory and fibrotic signaling pathways, highlighting additional therapeutic opportunities beyond the direct inhibition of TIMPs.

Despite this promising research on the involvement of TIMP-1 in fibrogenesis, some research provides conflicting data. Guenther et al. [45] demonstrated that although TIMP-1 is induced and upregulated during hepatic fibrogenesis, TIMP-1-deficient models remained capable of developing fibrosis. Similarly, Thiele et al. [46] reported that TIMP-1 was not essential for the progression of fibrogenesis in a comparable experimental setting. These findings highlight the functional redundancy among TIMP family members, which complicates efforts to isolate the specific contributions of TIMP-1 to fibrosis. Additionally, it may be possible that other compensatory mechanisms may become activated in TIMP-1-deficient mice. Consequently, targeting multiple TIMPs or combinatorial inhibitory approaches may be necessary to better elucidate their individual and collective roles in fibrogenesis.

Under different conditions, TIMP-1 may play a prominent anti-inflammatory role. Kamar et al. [47] measured MMP-9 and TIMP-1 levels in rats with MASLD treated with the drug telmisartan. Elevated MMP-9 levels were accompanied by increased signs of basement membrane disruption and inflammatory cell infiltration. Telmisartan treatment caused a significant increase in expression of TIMP-1 and a subsequent decrease in MMP-9. This resulted in less degradation of native membrane collagens and decreased inflammatory cell extravasation. While elevated TIMP-1 levels were measured in the context of reduced inflammation and immune cell infiltration, this study is unique in its approach to the early rather than established fibrosis. Their findings emphasize the importance of maintaining an appropriate MMP/TIMP balance. An early alteration in MMP activity within this fibrotic model led to pronounced inflammation rather than extracellular matrix accumulation and fibrosis.

TIMP-2 also plays a role in regulating other processes in the turnover of ECM. Benyon and Arthur [38] described the dual regulatory role of TIMP-2 in this context. At low concentrations, TIMP-2 enables pro-MMP-2 activation via cell-surface bridging as discussed earlier [6]. In contrast, at higher concentrations, excess TIMP-2 binds to active MMP-2 and inhibits its gelatinase activity, limiting ECM degradation. While TIMPs share several overlapping functions, important biochemical and spatial differences exist among family members. TIMP-2 and TIMP-3 exhibit weaker binding affinities for MMP-3 and MMP-7 compared with TIMP-1 [19]. Additionally, whereas TIMP-1 and TIMP-2 are soluble proteins secreted into the extracellular space, TIMP-3 is tightly bound to the ECM, conferring localized inhibitory activity [38]. Considering these differences, TIMPs appear to exhibit distinct temporal expression patterns during fibrogenesis. Al-Humadi et al. [7] reported that TIMP-1 levels were significantly elevated at days 30, 60, and 90 of treatment, whereas TIMP-2 levels significantly increased only at days 30 and 90. These findings suggest that, despite functional overlap, individual TIMPs may contribute to fibrogenesis at different stages of disease progression.

Given the consistent upregulation of TIMPs in fibrotic disease, TIMPs have been commonly used as biomarkers of liver fibrosis [43,44,48,49]. Hemmann et al. [9] demonstrated that hepatic TIMP-1 mRNA levels positively correlated with fibrosis severity, despite TIMP-1 expression alone being insufficient to induce fibrosis. These findings suggest that quantitative assessment of TIMP-1 expression may serve as a non-invasive surrogate marker of hepatic fibrotic burden. Indeed, TIMP-1 is incorporated into validated multi-analyte panels. For example, the Enhanced Liver Fibrosis (ELF) test (hyaluronic acid, TIMP-1, and PIIINP; AUROC ~0.83 for advanced fibrosis) and FibroSpect/FibroSURE-type assays (α2-macroglobulin, hyaluronic acid, and TIMP-1; AUROC ~0.86) have demonstrated superior sensitivity and specificity for advanced fibrosis compared with simple clinical scores such as FIB-4 and the NAFLD Fibrosis Score (NFS) [50,51].

While many functional aspects are common between TIMPs, it is also important to note that there are some key differences between each protein. For example, TIMP-1, perhaps the most well studied in fibrosis, is also the most limited in binding affinity when compared to the others, with a lower affinity for the membrane-bound MMPs (MMP-14, MMP-16, MMP24) and for MMP-19 [19].

Collectively, these findings indicate that TIMPs play a central role in both the development and maintenance of hepatic fibrosis. Sustained upregulation of TIMPs during fibrogenesis suggests that these proteins not only promote the survival of fibrogenic cells but also preserve the accumulated ECM by inhibiting MMP-mediated degradation. Consequently, effective antifibrotic strategies may require restoration of matrix turnover, potentially through the modulation or downregulation of TIMPs and other inhibitory regulators of MMP activity.

## 4. HSCs

HSCs are located in the space of Disse, positioned between hepatic sinusoids and hepatocytes, and constitute a minority of the total liver cell population [52]. Under physiological conditions, HSCs remain in a quiescent state, functioning primarily in vitamin A storage and ECM homeostasis. In response to acute or chronic hepatic injury, HSCs are activated by cytokines, chemokines, and growth factors secreted from damaged hepatocytes, cholangiocytes, liver sinusoidal endothelial cells, Kupffer cells, and other immune cells [53,54,55]. Activated HSCs are widely recognized as the predominant source of collagen deposition in advanced stages of hepatic fibrosis [56]. Other fibroblasts, such as PF or fibrocytes, share many attributes with the activated HSC and may contribute to early fibrotic remodeling [57]. During fibrosis resolution, activated HSCs are typically eliminated through apoptosis, cellular senescence, or reversion to a quiescent state [57,58].

In the activated state, HSCs not only directly contribute to the extracellular matrix by depositing collagen and other ECM proteins in their immediate area, but they become proliferative and contractile and can undergo chemotaxis, thereby amplifying fibrogenesis through both ECM synthesis and remodeling. This paradigm of myofibroblast activation and ECM deposition is conserved across multiple organ systems. Similar fibroblast populations have been identified in pulmonary fibrosis and myelofibrosis. This suggests that similar pathophysiology may be at play in diverse models of organ fibrosis.

Activated HSCs secrete TIMPs, which stabilize the fibrotic ECM by inhibiting MMPs that are responsible for degrading collagens, gelatins, and other ECM components. Huang et al. [42] demonstrated that activated HSCs secrete TIMP-1, α-SMA, FLI-1, and MCP-1, aligning with findings confirming the expression of these markers alongside collagen type I [35,44]. TIMP-1, specifically, exerts anti-apoptotic effects on HSCs classically via the CD63/β1-integrin complex [40,48]. Conversely, some MMPs have been recorded to encourage apoptosis in HSCs. Murphy et al. [40] showed that this effect could be mitigated with synthetic MMP inhibitors. Similarly, Murphy et al. [40], Hemmann et al. [9], and Birukawa et al. [59] have reported that MMP-2, MMP-9, MMP-14 may indirectly contribute to the resolution of fibrosis by inducing or accelerating HSC apoptosis, with inhibition of MMP-14 being executed through the use of TIMP-2. In addition to these, the apoptotic roles of other MMPs warrant further study. These results suggest that TIMPs inhibit apoptosis of activated HSCs by MMP inhibition, thereby facilitating the continued deposition of the fibrotic matrix.

Moreover, HSC activation may be driven by an influx of TIMPs released from inflamed or acutely injured cells. Ren et al. [48] found that HBV-infected hepatocytes from liver biopsies of both mice and human chronic HBV also begin to secrete elevated levels of TIMP-1 and incite significant collagen deposition in association with significant reductions in albumin levels. In response to this significant paracrine secretion of TIMP-1, there was activation of neighboring quiescent hepatocytes and HSCs, promoting fibrosis not only due to reduced viral clearance but also due to the effects of TIMPs modifying the microenvironment of chronic disease.

HSC activation is further modulated by mechanotransduction in response to matrix stiffness. Lachowski et al. [11] demonstrated that increased ECM rigidity downregulates MMP-9 expression while upregulating TIMP-1 secretion, thereby promoting collagen accumulation. Barberro-Becerra et al. [60] and Giraudi et al. [61] showed, using a simultaneous hepatocyte–HSC co-culture model, that HSCs exhibit increased expression of the activation/fibrotic markers α-SMA and TIMP-2 protein relative to HSCs cultured without hepatocytes, an effect dependent on cell-to-cell contact. However, it is well established that HSCs can also spontaneously activate and express these markers when cultured alone on plastic dishes, independent of other cell types [62]. This indicates that, while paracrine and juxtacrine signaling from hepatocytes and other liver cells can further modulate or amplify HSC activation, such feedback is not strictly required for the upregulation of fibrotic morphology and marker expression. Further studies may be necessary to confirm whether the regulation of various MMPs and other proteins are similarly influenced by the presence of signaling from other cells.

Current data make it clear that activated HSCs play a key role in the development and maintenance of liver fibrosis. HSC production of matrix participates in the buildup of fibrotic scar tissue by depositing excess collagen and enhancing secretion of TIMPs.

## 5. Regulation of Hepatic Fibrosis by the MMP/TIMP Balance

The preceding sections establish that individual MMPs and TIMPs exert diverse, often opposing, effects on the fibrotic matrix. The central determinant of whether chronic injury progresses to cirrhosis or resolves, however, is not the absolute abundance of any single enzyme or inhibitor but the dynamic equilibrium between proteolytic MMP activity and TIMP-mediated inhibition. Under physiological conditions, MMPs and TIMPs act in concert to maintain ECM homeostasis: MMPs continuously degrade and remodel matrix to permit reliable turnover, while TIMPs constrain that proteolysis so that it does not damage healthy parenchyma. Disruption of this equilibrium, through either reduced MMP activity or TIMP overexpression, shifts the ECM from a physiological to a pathological state and permits the unopposed accumulation of fibrillar collagen that underlies progressive fibrosis [63,64]. Framing fibrosis as a disorder of matrix turnover balance, rather than of matrix synthesis alone, reorients the therapeutic question from “how is collagen made?” to “why is collagen not degraded?”. It is this reframing that motivates the molecular strategies discussed below.

**Figure 2:**
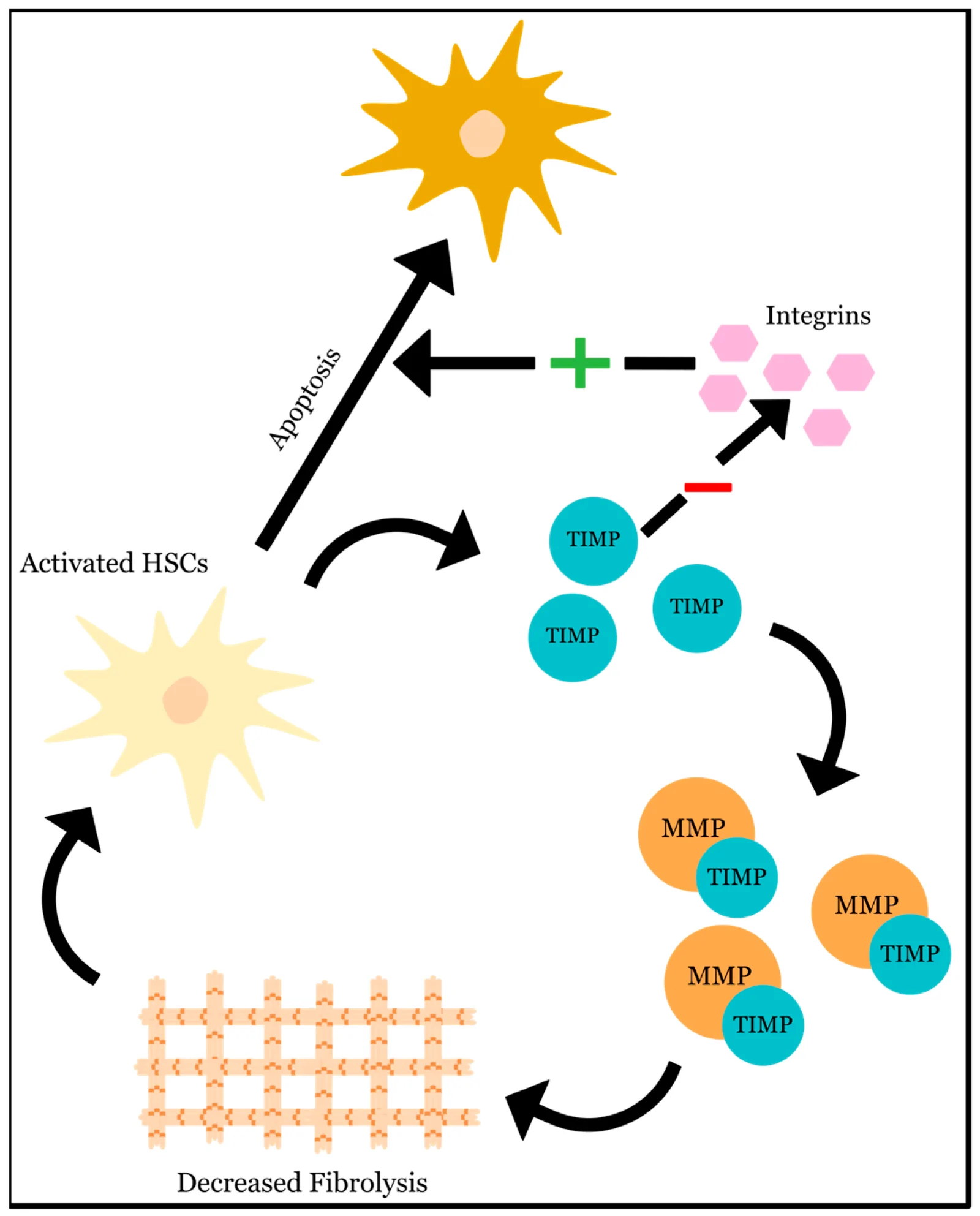
Proposed positive feedback loop governing hepatic stellate cell (HSC) activation and matrix accumulation. Activated HSCs secrete elevated levels of TIMPs, which exert dual regulatory effects: (1) direct 1:1 stoichiometric inhibition of active MMPs, leading to suppressed ECM degradation (fibrolysis) and structural matrix accumulation, and (2) inhibition of apoptotic signaling pathways via integrin-mediated mechanotransduction. Persistent matrix stiffness and reduced apoptosis sustain the pool of activated fibroblastic HSCs, perpetuating a self-reinforcing fibrogenic loop. The green plus sign indicates pathway activation/survival; the red bar indicates pathway inhibition [3,4,5,6].

The MMP/TIMP imbalance in established fibrosis is not a static endpoint but a self-perpetuating circuit that couples biochemical and mechanical signals within activated HSCs (see Figure 2). Activated HSCs deposit fibrillar collagen and simultaneously secrete TIMPs, which suppress MMP-mediated degradation of that collagen. The resulting matrix accumulation increases tissue stiffness, and this rising rigidity feeds back onto the cells through mechanotransduction: Lachowski et al. [11] demonstrated that increased matrix stiffness downregulates MMP-9 activity while upregulating TIMP-1 secretion in HSCs, whereas TIMP-1 knockdown restores MMP-9 activity. Stiffness therefore begets further TIMP dominance, which results in further matrix retention and further stiffening, a positive feedback loop that stabilizes the fibrotic niche independently of the initiating insult. Two additional circuits reinforce this loop. First, TIMP-1 directly prolongs the lifespan of the cells driving the process: it inhibits apoptosis of activated HSCs through effects on MMP inhibition, classically via the CD63/β1-integrin complex, thereby preserving the fibrogenic cell population that sustains matrix deposition [40]. Second, TIMP-1 amplifies inflammatory recruitment by suppressing microRNA-145 in activated HSCs, relieving repression of Fli-1 and increasing MCP-1 secretion, which recruits monocytes and macrophages that in turn secrete profibrotic cytokines such as TGF-β [42]. Consequently, targeting the TIMP-1/miRNA-145/MCP-1 axis represents a promising strategy to interrupt this inflammatory driver [42]. The equilibrium is thus maintained by interlocking the mechanical, survival, and inflammatory feedback arms, which explains both the self-sustaining character of advanced fibrosis and why interventions targeting a single molecule are unlikely to be successful.

Just as the balance is maintained spatially, it also evolves temporally through the natural history of injury, and interpreting isolated expression measurements without reference to disease phase can be misleading. In acute injury, MMP expression is induced early, clearing cellular debris and degrading matrix to create space for regeneration, with MMP-2 and MMP-9 upregulated during this initial phase. At this stage a controlled rise in TIMP-1 may be protective rather than pathological: in an early MASLD model, telmisartan-induced elevation of TIMP-1 reduced MMP-9-mediated basement-membrane disruption and limited inflammatory cell infiltration, indicating that appropriate MMP restraint early in injury can attenuate inflammation rather than drive matrix accumulation [47]. As injury becomes chronic and fibrosis progresses, sustained TIMP upregulation shifts the balance toward matrix stabilization. Global hepatic MMP activity declines even where cellular MMP secretion persists, and MMP-9 in particular exhibits biphasic behavior. Early induction is followed by later downregulation concurrent with TIMP elevation so that insufficient ECM degradation permits progressive fibrotic accumulation. TIMP-1 and TIMP-2 also display distinct temporal kinetics during progression, suggesting that individual family members contribute at different disease stages rather than uniformly throughout. When the initiating insult is removed and resolution begins, the balance can tip back toward degradation. Several MMPs are upregulated during resolution, and this upregulation is meaningful only relative to concurrent TIMP expression. Restorative macrophages accumulate after injury cessation and secrete MMP-12 and MMP-13 while promoting HSC apoptosis, supporting a pro-resolutive role for these enzymes [10]. Effective fibrolysis therefore requires sufficient MMP activity to overcome TIMP-mediated inhibition and to eliminate the fibrogenic HSC population, not merely a rise in MMP abundance in absolute terms. The goal is not to maximize MMP activity, nor to minimize TIMP activity at all times, but rather to restore the phase-appropriate equilibrium, preserving protective early restraint while re-enabling degradation during the resolution window.

The temporal and feedback-driven nature of the MMP/TIMP balance explains why historical broad-spectrum enzyme modulation has proven clinically ineffective. Non-selective, sustained MMP modulation ignores the biphasic and cell-specific roles of individual proteases, suppressing pro-resolution MMP-12/-13 activity as readily as pro-fibrotic activity, and disregarding the disease phase in which intervention occurs. Because the pathological equilibrium is maintained by redundant, interlocking feedback arms, indiscriminate targeting of a single enzyme class is ineffective. This is the same redundancy reflected in the retained fibrogenic capacity of TIMP-1-deficient models and in the overlapping inhibitory profiles across the TIMP family. Xie et al. [63] demonstrated that inhibiting TGF-β/Smad signaling (including key components such as TGF-β1, TGF-βRI, TGF-βRII, and Smad2/3/7) coupled with upregulating the MMP-2/TIMP-1 ratio reversed hepatic fibrosis, implicating MMP/TIMP regulation, rather than absolute MMP or TIMP abundance, as the tractable therapeutic axis for controlling ECM remodeling [64]. Reversing this imbalance to promote ECM degradation can be achieved by either enhancing MMP expression or inhibiting TIMP activity [65]. Approaches include gene therapy and vector-mediated approaches targeting TIMP expression to reduce TIMP protein levels and enhance MMP activity in preclinical models [66,67]. The clinical relevance of this axis is reinforced by human data. Polymorphisms in the TIMP-1 and TIMP-2 genes are associated with the rate of fibrosis progression in patients with chronic hepatitis C, indicating that inter-individual variation in the inhibitors helps determine outcome [64]. The same shift in matrix-turnover balance is increasingly measurable in patients, as circulating and imaging-based surrogates of ECM remodeling now permit noninvasive tracking of fibrosis and its regression [65]. Because the fibrotic state is stabilized by interlocking mechanical, survival, and inflammatory components and shifts across disease phase, regression requires interrupting the self-reinforcing loop at more than one node and re-enabling degradation during the resolution window.

## 6. Conclusions

It is clear that the interplay between MMPs and TIMPs secreted by HSCs and other fibroblasts is crucial in the development and maintenance of liver fibrosis. Activated HSCs are the main cellular source of scar tissue and fibrogenesis and are known to secrete MMPs and TIMPs. MMPs have been shown to have diverse effects in both their upregulation during the initiation of fibrosis and their downregulation during subsequent fibrosis. The eventual repair of fibrosis seems to require an increase in MMP expression to cleave the matrix of the scar tissue and rectify this part of the system. In addition, MMPs may be crucial in facilitating the apoptosis of activated HSCs and decreasing the rate at which fibrotic ECM is deposited. In opposition to these MMPs, TIMPs maintain the fibrotic state by prolonging the lifespans of the activated HSCs.

More research is required to identify therapeutic interventions for inhibiting TIMPs and reactivating various MMPs. Emerging strategies include the development of small-molecule inhibitors that selectively target TIMP-MMP interactions, antisense oligonucleotides (ASOs) designed to silence TIMP-1 and TIMP-2 gene expression at the post-transcriptional level, and RNA interference (RNAi)-based approaches using small interfering RNAs (siRNAs) delivered via nanoparticle or viral vector systems to suppress TIMP production in activated HSCs.

Beyond gene silencing, epigenetic editing tools, such as CRISPR-dCas9 fused to epigenetic modifiers, offer the potential to reprogram the chromatin landscape at TIMP and MMP gene loci, restoring a pro-fibrolytic MMP/TIMP balance without permanent genomic alteration. Additionally, cell-specific delivery platforms, including HSC-targeted lipid nanoparticles and exosome-based carriers, are being explored to enhance the precision and safety of these interventions by confining their effects to the fibrotic microenvironment. Such strategies extend beyond the direct inhibition of TIMPs and include the targeting of upstream mediators, such as SYK, to facilitate MMP reactivation and confer the benefits of TIMP suppression while broadly attenuating fibrotic and inflammatory secretory responses. Collectively, these approaches represent a paradigm shift from broad-spectrum antifibrotic therapies toward molecularly targeted strategies that aim to restore the physiological MMP/TIMP equilibrium and promote fibrosis regression.

## Figures and Tables

**Table 1:** Inhibitory Affinities and Substrate Specificities of TIMPs across the MMP Family.

MMP	Primary Function(s)	TIMP-1	TIMP-2	TIMP-3	TIMP-4	References
MMP-1	Collagens I, II, III, VII, X; gelatin; tenascin; perlecan; entactin; aggrecan; proTGF-α; proHB-EGF; PAR1	++	(+)	+	(+)	[20,21]
MMP-2	Gelatin; collagens I, II, III, IV, V, X, XI; fibronectin; laminin; elastin; vitronectin; TGFβ-LAP	++	+++	++	+	[20,21,22,23,24,25]
MMP-3	Collagens III, IV, V, IX, X; aggrecan; decorin; fibronectin; laminin; elastin; proteoglycans; E-cadherin; proMMP-1, -9; VEGF	++	(+)	++	(+)	[20,21,22,26]
MMP-7	Collagens I, IV; gelatin; elastin; fibronectin; laminin; vitronectin; tenascin; decorin; proteoglycans; E-cadherin; RANKL; proHB-EGF	++	(+)	(+)	(+)	[20,22]
MMP-8	Collagens I, II, III; gelatin; aggrecan; laminin α5 (LM-511)	++	(+)	(+)	(+)	[20,22]
MMP-9	Gelatin; collagen IV, V; elastin; entactin; aggrecan; vitronectin; laminin-111; fibronectin; TGFβ-LAP; VEGF; IL-8; E-cadherin	++	+	(+)	(+)	[20,22,27]
MMP-10	Collagens III, IV, V; aggrecan; fibronectin; laminin; proteoglycans; proMMP-1	(+)	(+)	(+)	(+)	[20]
MMP-11	Collagen IV; gelatin; fibronectin; laminin; IGFBP-1; α1-antitrypsin	(+)	(+)	(+)	(+)	[9,22]
MMP-12	Elastin; collagens I, IV, V; gelatin; casein; fibronectin; vitronectin; laminin; entactin; fibrinogen; osteonectin; aggrecan; myelin; proteoglycans; α1-antitrypsin	(+)	(+)	(+)	(+)	[20,22]
MMP-13	Collagens I, II, III, IV, IX, X, XIV; gelatin; tenascin-C; aggrecan; perlecan; fibronectin; osteonectin; laminin; fibrillin-1; plasminogen; proMMP-2, -9	(+)	(+)	++	(+)	[21,28,29]
MMP-14	Collagens I, II, III; gelatin; fibronectin; laminin-332; fibrin; aggrecan; vitronectin; tenascin; proteoglycans; fibrillin-1; thrombospondin-1; TGFβ-LAP; RANKL; EphA2; proMMP-2, -13	−	++	(+)	+	[18,20,23,24,26,30,31]
MMP-15	Fibronectin; laminin; aggrecan; gelatin; vitronectin; entactin/nidogen; tenascin; perlecan; fibrin; collagen IV; proMMP-2, -13	−	(+)	(+)	(+)	[20,30,31]
MMP-16	Collagen III; collagens I, II, IV; gelatin; casein; fibronectin; laminin; fibrin; proMMP-2, -9	−	(+)	(+)	(+)	[20,23,30,31]
MMP-17	Gelatin; fibrinogen; fibrin; proMMP-2	(+)	(+)	(+)	(+)	[20,30]
MMP-19	Collagens I, IV; gelatin; fibronectin; laminin; nidogen; tenascin-C; entactin; aggrecan; proMMP-9	−	(+)	(+)	(+)	[20,30,31]
MMP-20	Amelogenin; aggrecan	(+)	(+)	(+)	(+)	[20,22]
MMP-21	No well-defined ECM substrate identified to date	(+)	(+)	(+)	(+)	[20,22]
MMP-23	Gelatin	(+)	(+)	(+)	(+)	[20,22]
MMP-24	Fibronectin; gelatin; proteoglycans; proMMP-2	−	(+)	(+)	(+)	[20,30,31]
MMP-25	Collagen IV; gelatin; fibronectin; proteoglycans; proMMP-2	(+)	(+)	(+)	(+)	[20,30]
MMP-26	Collagen IV; fibrinogen; fibronectin; gelatin; vitronectin; β-casein; α1-antitrypsin; IGFBP-1; proMMP-9	(+)	(+)	(+)	(+)	[20,22]
MMP-27	Gelatin	(+)	(+)	(+)	(+)	[20,22]
MMP-28	Casein	(+)	(+)	(+)	(+)	[20,22]

Legend: +++ = Exceptionally tight binding (femtomolar range, ~10^−15^ M; e.g., TIMP-2/MMP-2 ~0.6 fM). ++ = Very high affinity (K_i_/K_d_ ≤ ~1 nM; picomolar range). + = Moderate/effective affinity (K_i_/K_d_ ~1–100 nM). (+) = Presumed inhibition; broad-spectrum MMP inhibition expected, but specific quantitative constants were not identified in the retrieved literature [22]. − = Documented poor or negligible inhibition (K_i_/K_d_ ≥ ~100 nM). Note: Binding affinities are ordinal categories based on published inhibition constants (K_i_) or dissociation constants (K_d_) from enzymatic kinetic and biophysical studies (e.g., progress-curve/Morrison, SPR, BLI, ITC). Because values derive from varying experimental methods and MMP constructs (full-length vs. catalytic domain), tiers represent approximate ordinal categories rather than precise quantitative thresholds. Disclaimer: Primary Function(s) represents a simplified summary for orientation and is not an exhaustive account of each MMP’s substrate repertoire or biological roles, as most MMPs cleave numerous additional ECM and non-ECM substrates beyond those listed [6,32,33,34]. Abbreviations: ADAM, A disintegrin and metalloproteinase; BLI, bio-layer interferometry; ECM, extracellular matrix; ITC, isothermal titration calorimetry; K_d_, dissociation constant; K_i_, inhibition constant; MMP, matrix metalloproteinase; MT-MMP, membrane-type matrix metalloproteinase; PAR1, protease-activated receptor 1; RANKL, receptor activator of nuclear factor-κB ligand; SPR, surface plasmon resonance; TGF, transforming growth factor; TIMP, tissue inhibitor of metalloproteinases; VEGF, vascular endothelial growth factor.

## Data Availability

No new data were created or analyzed in this study. Data sharing is not applicable to this article.
